# A General Principle of Neuronal Evolution Reveals a Human-Accelerated Neuron Type Potentially Underlying the High Prevalence of Autism in Humans

**DOI:** 10.1093/molbev/msaf189

**Published:** 2025-09-05

**Authors:** Alexander L Starr, Hunter B Fraser

**Affiliations:** Department of Biology, Stanford University, Stanford, CA 94305, USA; Department of Biology, Stanford University, Stanford, CA 94305, USA

**Keywords:** evolution, brain, gene expression, autism, human

## Abstract

The remarkable ability of a single genome sequence to encode a diverse collection of distinct cell types, including the thousands of cell types found in the mammalian brain, is a key characteristic of multicellular life. While it has been observed that some cell types are far more evolutionarily conserved than others, the factors driving these differences in the evolutionary rate remain unknown. Here, we hypothesized that highly abundant neuronal cell types may be under greater selective constraint than rarer neuronal types, leading to variation in their rates of evolution. To test this, we leveraged recently published cross-species single-nucleus RNA-sequencing datasets from three distinct regions of the mammalian neocortex. We found a strikingly consistent relationship where more abundant neuronal subtypes show greater gene expression conservation between species, which replicated across three independent datasets covering >10^6^ neurons from six species. Based on this principle, we discovered that the most abundant type of neocortical neurons—layer 2/3 intratelencephalic excitatory neurons—has evolved exceptionally quickly in the human lineage compared to other apes. Surprisingly, this accelerated evolution was accompanied by the dramatic down-regulation of autism-associated genes, which was likely driven by polygenic positive selection specific to the human lineage. In summary, we introduce a general principle governing neuronal evolution and suggest that the exceptionally high prevalence of autism in humans may be a direct result of natural selection for lower expression of a suite of genes that conferred a fitness benefit to our ancestors while also rendering an abundant class of neurons more sensitive to perturbation.

## Introduction

With the advent of single-cell RNA sequencing (scRNA-seq), it became possible to systematically delineate molecularly defined cell types across the brain (Zeisel et al. 2015; Tasic et al. 2016). As more large-scale datasets were published, it quickly became clear that the mammalian brain contains a staggering array of neuronal cell types, with recent whole-brain studies identifying nearly as many neuronal types as there are protein-coding genes in the genome (Zeisel et al. 2015 ; Tasic et al. 2016; Yao et al. 2023). In addition, cross-species atlases in the neocortex revealed that most cortical neuronal types are highly conserved in primates and rodents, with very few neocortical neuronal types being specific to primates and none being entirely specific to humans (Hodge et al. 2019; Krienen et al. 2020; Bakken et al. 2021a , 2021b; Ma et al. 2022; Jorstad et al. 2023). This suggests that divergence involving homologous cell types—such as their patterns of gene expression, relative proportions, and connectivity—may play a central role in establishing uniquely human cognition.

Two decades before the generation of these cross-species cell type atlases, the first whole-genome sequences of eukaryotes were published, enabling genome-wide studies of evolution for the first time (Eyre-Walker 1999). One of the first questions to be addressed in the nascent field of evolutionary genomics was why some proteins are highly conserved throughout the tree of life, whereas, others evolve so quickly as to be almost unrecognizable as orthologs even over relatively short divergence times (Duret and Mouchiroud 2000; Hirsh and Fraser 2001; Pál et al. 2001; Fraser et al. 2002). A protein's expression level emerged as the strongest and most universal predictor of its evolutionary rate, with highly expressed proteins accumulating fewer protein-coding changes due to greater constraint (Pál et al. 2001; Drummond et al. 2005, 2006; Drummond and Wilke 2008).

In contrast to tens of thousands of publications about the evolutionary rates of proteins (Yang 2007), the evolutionary rates of cell types, another key building block of multicellular life, have received relatively little attention (Arendt et al. 2016). Just as different proteins make up every cell, different cell types make up every multicellular organism. Furthermore, just as protein evolutionary rates are measured by the total rate of change of their amino acids, the evolutionary rates of cell types—which are typically defined by their patterns of gene expression—can be measured by divergence in genome-wide gene expression (Hodge et al. 2019; Krienen et al. 2020; Bakken et al. 2021a , 2021b; Ma et al. 2022; Jorstad et al. 2023). For example, it is well-established that the gene expression in neurons is more conserved between humans and mice than the gene expression in glial cell types, such as astrocytes, oligodendrocytes, and microglia (Pembroke et al. 2021). Previous analogies between genes and neural cell types have been fruitful for understanding the evolution of novel cell types (Tosches et al. 2018; Hodge et al. 2019; Peng et al. 2019; Kebschull et al. 2020; Luo 2021), providing an encouraging precedent for our analogy.

One area that has been explored more thoroughly is the association of specific cell types with human diseases and disorders (Jagadeesh et al. 2022). For example, integration of gene-trait associations with cell type-specific expression profiles has revealed that microglia likely play a central role in Alzheimer's disease (Jansen et al. 2019; Wightman et al. 2021). Similar analyses have also revealed that layer 2/3 intratelencephalic excitatory (L2/3 IT neurons)—which enable communication between neocortical areas (Galakhova et al. 2022) and are thought to be important for uniquely human cognitive abilities (Berg et al. 2021; Galakhova et al. 2022)—likely play a particularly important role in autism spectrum disorder (ASD) and schizophrenia (SCZ) (Parikshak et al. 2013; Kanton et al. 2019; Velmeshev et al. 2019; Batiuk et al. 2022; Trubetskoy et al. 2022; Pintacuda et al. 2023; Dear et al. 2024; Wamsley et al. 2024), together with deep layer IT neurons (Trubetskoy et al. 2022; Ruzicka et al. 2024; Sullivan et al. 2024). For example, a recent large-scale single-cell RNA-sequencing study found that L2/3 IT neurons and Somatostatin+ (SST+) inhibitory neurons were the most affected in people with ASD (Wamsley et al. 2024). In another study, proteins that interact with ASD-linked proteins were strongly and specifically enriched for differential expression between ASD cases and controls in L2/3 IT neurons (Pintacuda et al. 2023). Collectively, these and other results point to a key role for L2/3 IT neurons in ASD, though other populations of neurons likely also play important roles. ASD and SCZ are neurodevelopmental disorders with different but overlapping characteristics, including major effects on social behavior (Dodell-Feder et al. 2015; Jutla et al. 2022; Sato et al. 2023). Interestingly, individuals with ASD are more likely to be diagnosed with SCZ than individuals without an ASD diagnosis (Lugo Marín et al. 2018; Lai et al. 2019; Zheng et al. 2021; Jutla et al. 2022). Furthermore, there is a strong overlap in the genes that have been implicated in both disorders (Jutla et al. 2022; Trubetskoy et al. 2022).

From an evolutionary perspective, it has been proposed that ASD and SCZ may be unique to humans (Crow 1997; Sikela and Searles Quick 2018; Zug and Uller 2022 ). This is primarily based on two main lines of reasoning. First, ASD- and SCZ-associated behaviors that could reasonably be observed in non-human primates (e.g. SCZ-associated psychosis) have been observed either infrequently or not at all in non-human primates (Crow 1997). However, ASD-like behavior has been observed in non-human primates (Yoshida et al. 2016) and the difficulties inherent to cross-species behavioral comparisons, combined with relatively low sample sizes, make it difficult to compare the prevalence of these behaviors in human and non-human primate populations. Second, core ASD- and SCZ-associated behavioral differences involve cognitive traits that are either unique to or greatly expanded in humans (e.g. speech production and comprehension or theory of mind) (Marrus et al. 2011; Mody and Belliveau 2013; Faughn et al. 2015; MacLean 2016; Chang et al. 2022). As a result, certain aspects of ASD and SCZ are inherently unique to humans.

While comparing interindividual behavioral differences across species remains challenging, recent molecular and connectomic evidence lend credence to the idea that the incidence of ASD and SCZ increased during human evolution. For example, large-scale sequencing studies in both ASD and SCZ cohorts have identified an excess of genetic variants in human-accelerated regions (HARs)—genomic elements that were largely conserved throughout mammalian evolution but evolved rapidly in the human lineage (Pollard et al. 2006; Doan et al. 2016; Shin et al. 2024). Furthermore, transcriptomic studies have identified a human-specific shift in the expression of some synaptic genes during development that is disrupted in ASD (Liu et al. 2016). In addition, connectomic studies have shown that human–chimpanzee divergence in brain connectivity overlaps strongly with differences between humans with and without SCZ (van den Heuvel et al. 2019). Overall, evidence suggests that ASD and SCZ may be particularly prevalent in humans, but the factors underlying this increased prevalence remain unknown. Positive selection—also known as adaptive evolution—of brain-related traits in the human lineage has been proposed to underlie this increase (Crow 1997; Burns 2004; Ploeger and Galis 2011; Sikela and Searles Quick 2018; Zug and Uller 2022). Although this idea is supported by the links between HARs (many of which are thought to have been positively selected [Pollard et al. 2006]) and ASD and SCZ, there is no direct evidence for positive selection on the expression of genes linked to ASD and SCZ.

Here, we set out to test whether the inverse relationship between the abundance and evolutionary rates—which has been well-established for proteins (Pál et al. 2001; Drummond et al. 2005, 2006; Drummond and Wilke 2008)—might also hold for cell types. We found a robust negative correlation between the cell type proportion and the evolutionary divergence in the neocortex, suggesting that this relationship holds at multiple levels of biological organization. Based on this, we identify unexpectedly rapid evolution of L2/3 IT neurons and strong evidence for polygenic positive selection for reduced expression of ASD-linked genes in the human lineage, suggesting that positive selection may have increased the prevalence of ASD in modern humans.

## Results

### Cell Type Proportion as a General Factor Governing the Rate of Neuronal Evolution

**Fig. 1.**
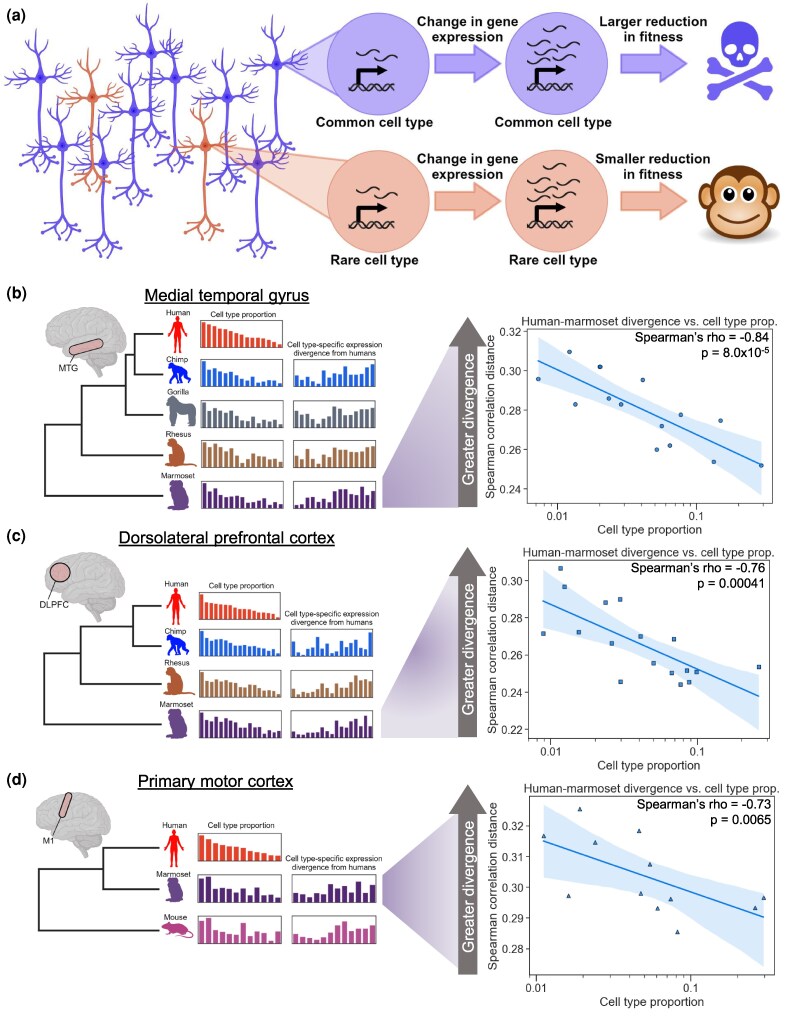
More common neuronal cell types evolve more slowly than rare types. a) Rationale for the hypothesis that more common neuronal types might evolve more slowly than rarer types. A gene expression change in a common cell type has a large negative effect on fitness, whereas the same change in a rarer cell type has a smaller effect. Made with BioRender. b) Left: outline of our data analysis strategy. SnRNA-seq from the MTG of five species (14 subclasses of neurons) was used to estimate each cell type's proportion and pairwise divergence between species. Right: plot showing the correlation between the neuronal subclass proportion (log_10_ scale on the *x*-axis) and the subclass-specific divergence between the human and the marmoset in the MTG. A representative iteration from 100 independent down-samplings is shown. Spearman's ρ and *P*-value shown are the median across 100 independent down-samplings (see Materials and Methods for details). The line and shaded region are the line of best fit from a linear regression and 95% confidence interval, respectively. c) Same as b), but snRNA-seq from the DLPFC (17 subclasses of neurons) of four species was analyzed. d) Same as b), but snRNA-seq from M1 (12 subclasses of neurons) of three species was analyzed.

Based on the gene-cell type analogy outlined earlier, we hypothesized that a change in the gene expression in a more abundant cell type may tend to have more negative fitness effects than the same change in a less abundant cell type (Fig. 1a). If this were the case, this would lead to greater selective constraint, and thus slower divergence, of global gene expression in more abundant cell types.

Testing this hypothesis requires comparing two quantities: the cell type proportions and the evolutionary divergence in genome-wide gene expression levels between orthologous cell types across species. Importantly, both quantities can be estimated from the same single-nucleus RNA-seq (snRNA-seq) data, facilitating comparison between them. To ensure sufficient statistical power, we searched the literature for published snRNA-seq datasets that fulfilled a stringent pair of criteria. First, they must have multiple species profiled in the same study using the same snRNA-seq protocols for each species within a study. Second, they must contain at least 10 orthologous cell types having 250 or more cells per species (not including immune cells, as these do not have stable cell type proportions). We identified three studies fulfilling these criteria, focused on three distinct regions of the mammalian neocortex: medial temporal gyrus (MTG), dorsolateral prefrontal cortex (DLPFC), and primary motor cortex (M1) (Bakken et al. 2021a; Ma et al. 2022; Jorstad et al. 2023 ). All three studies included samples from 3 to 5 species, including human and marmoset, with 300,000 to 500,000 neuronal nuclei profiled per study (Bakken et al. 2021a; Ma et al. 2022; Jorstad et al. 2023). These nuclei were clustered into between 12 and 17 neuronal subclasses (with at least 250 cells per species) in each study, which we then used for our analyses (Bakken et al. 2021a; Ma et al. 2022; Jorstad et al. 2023). Throughout, we use the term cell type for the general concept of different types of cells and as an umbrella term for both subclasses and subtypes, use the term subclass for the traditional classification of neuronal types found in the neocortex, and reserve the term subtype for more fine-grained clustering of cells.

To test our hypothesis, we began by comparing human and marmoset (the only pair of species present in all three datasets) in the MTG, which had the greatest sequencing depth. We first estimated gene expression divergence for each of 14 subclasses using the Spearman correlation distance (1—Spearman's ρ) between the pseudobulked expression of each species for each neuron subclass, restricting to one-to-one orthologous genes (see Materials and Methods). We observed a surprisingly strong negative correlation between the subclass proportion and the gene expression divergence (Spearman's ρ = −0.84, *P* = 8.0×10^−5^, Fig. 1b), indicating that more abundant neuronal subclasses showed greater conservation of genome-wide gene expression. To ensure that estimates of cell type-specific expression divergence were not biased by the cell type proportion itself, we analyzed the same number of cells and total reads for each cell type in each species. Specifically, for all analyses, we report the median ρ and *P*-values from 100 independent down-samplings of cells and pseudobulked counts without replacement (see Materials and Methods).

We next asked whether the same pattern was present in the other cortical regions. We observed a similar strong negative correlation in the two other independently generated datasets (Spearman's ρ = −0.76, *P* = 0.00041 in the DLPFC, Fig. 1c; Spearman's ρ = −0.73, *P* = 0.0065 in the M1, Fig. 1d). This replication suggests that the relationship we observed holds true across the primate neocortex. In addition, the fact that methodological details and biological samples differ across these studies lends additional robustness to any patterns shared by all three.

**Fig. 2.**
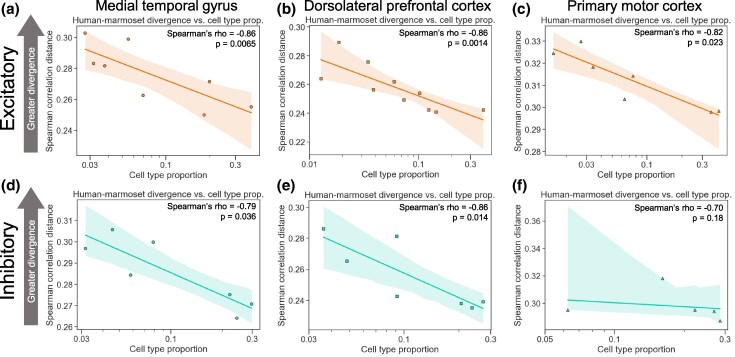
More common neuronal cell types evolve more slowly than rare types within excitatory and inhibitory classes. a) Plot showing the correlation between the neuronal subclass proportion (log_10_ scale on the *x*-axis) and the subclass-specific divergence between the human and the marmoset for excitatory neurons in the MTG. A representative iteration from 100 independent down-samplings is shown. Spearman's ρ and *P*-value shown are the median across 100 independent down-samplings (see Materials and Methods for details). The line and shaded region are the line of best fit from a linear regression and 95% confidence interval, respectively. b) Same as in a) but for the DLPFC data. c) Same as in a) but for the M1 data. d) Same as in a) but for inhibitory neurons. e) Same as in b) but for inhibitory neurons. f) Same as in c), but for inhibitory neurons.

To explore the generality of this result in additional species, we repeated this analysis between every pair of species in each dataset. We observed similarly strong negative correlations across all pairwise comparisons (supplementary figs. S1 to S3, Supplementary Material online), with the interesting exception of comparisons between humans and non-human great apes, where a weaker negative correlation was observed (discussed below). Furthermore, we observed strong negative correlations within excitatory or inhibitory subclasses in all three brain regions (Fig. 2, supplementary figs. S4 to S9, Supplementary Material online, although this correlation does not reach statistical significance for inhibitory neurons in M1, potentially due to having only five subclasses in that dataset). In addition, we tested all possible combinations of a wide variety of filtering parameters, analysis decisions, and distance metrics, finding that this negative correlation was generally robust to any reasonable choice of parameters we made (supplementary table S1, Supplementary Material online).

**Fig. 3.**
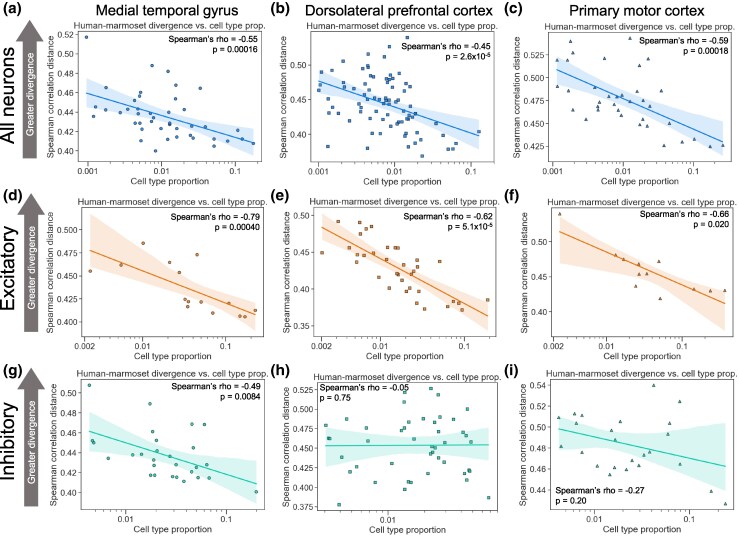
More common neuronal cell types evolve more slowly than rarer types at the subtype level. a) Plot showing the correlation between the neuronal subtype proportion (log_10_ scale on the *x*-axis) and the subtype-specific divergence between the human and the marmoset in the MTG. A representative iteration from 100 independent down-samplings is shown. Spearman's ρ and *P*-value shown are the median across 100 independent down-samplings (see Materials and Methods for details). The line and shaded region are the line of best fit from a linear regression and 95% confidence interval, respectively. b) Same as in a) but for the DLPFC data. c) Same as in a) but for the M1 data. d) Same as in a) but for excitatory neurons. e) Same as in b) but for excitatory neurons. f) Same as in c), but for excitatory neurons. g) Same as in a) but for inhibitory neurons. h) Same as in b) but for inhibitory neurons. i) Same as in c) but for inhibitory neurons.

Next, we investigated this relationship at the level of neuronal subtypes, a finer-grained clustering with ∼4-fold more cell subtypes than subclasses. We found strong negative correlations between the subtype proportion and the expression divergence when using all neurons (Fig. 3a–c, supplementary figs. S10 to S12, Supplementary Material online) or only excitatory neurons (Fig. 3d–f, supplementary figs. S13 to S15, Supplementary Material online). When restricting our analysis to inhibitory neurons, this correlation was statistically significant in the MTG and in two of three comparisons (mouse–marmoset and human–mouse) in the M1, but not in the DLPFC (Fig. 3g–i, supplementary figs. S16 to S18, Supplementary Material online). This may reflect the lower read depth (average of 180,054 counts used for the DLPFC, compared to 254,703 for the M1 and 325,422 for the MTG) or lower numbers of cells per subtype in the DLPFC data compared to the other datasets, as we observed a much stronger negative correlation (Spearman's ρ = −0.50, *P* = 0.057) when restricting to subtypes with at least 500 cells in the DLPFC data (supplementary fig. 19, Supplementary Material online). Overall, our results suggest that there is a strong, robust negative correlation between the expression divergence and the cell type proportion for neocortical neurons.

**Fig. 4.**
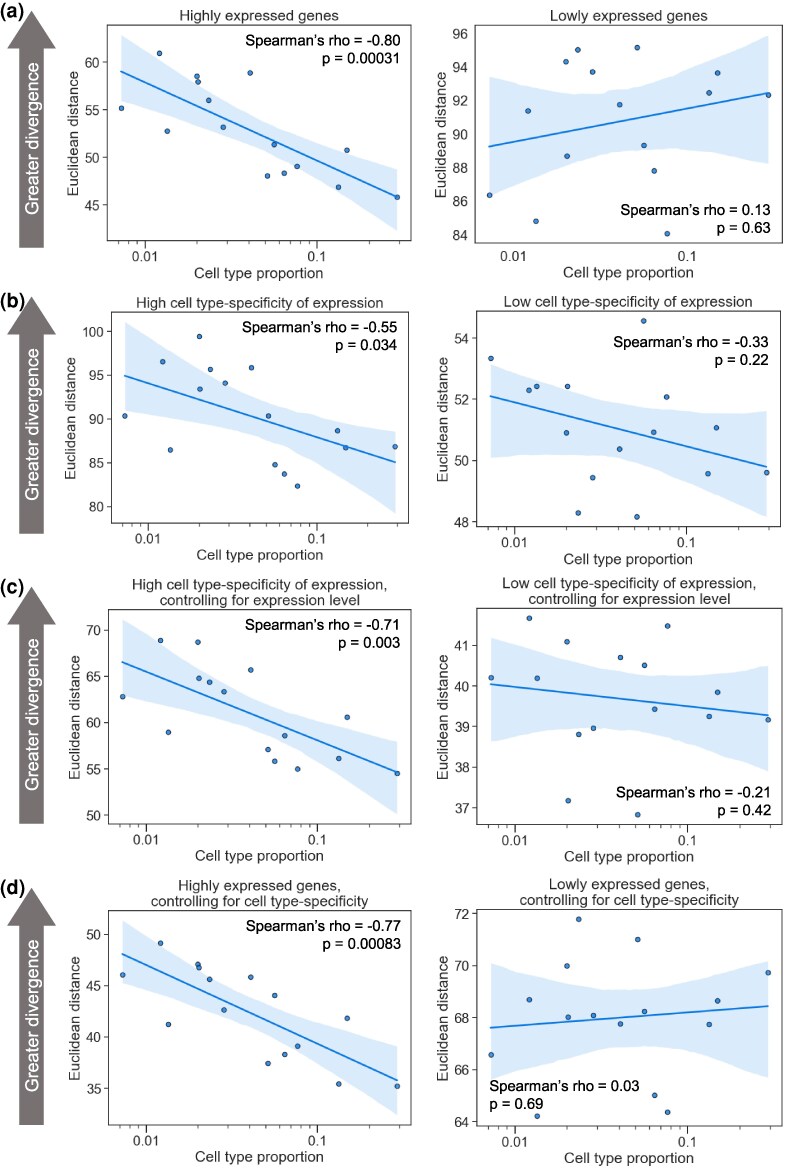
More highly expressed, cell type-specific genes drive the negative correlation between the cell type proportion and the evolutionary divergence. a) Left: Plot showing the correlation between the neuronal subtype proportion (log_10_ scale on the *x*-axis) and the subtype-specific divergence for highly expressed genes between the human and the marmoset in the MTG. A representative iteration from 100 independent down-samplings is shown. Spearman's ρ and *P*-value shown are the median across 100 independent down-samplings (see Materials and Methods for details). The line and shaded region are the line of best fit from a linear regression and 95% confidence interval, respectively. Right: Same as the left, but for lowly expressed genes. b) Left: Same as in a) but for genes with more cell type-specific expression; Right: Same as left but for genes with less cell type-specific expression. c) Same as in b) but controlling for expression level (see Materials and Methods). d) Same as in a) but controlling for cell type-specificity of expression (see Materials and Methods).

Finally, we investigated the properties of the genes driving the negative correlation we observed. First, we stratified genes into three equally sized bins by their expression level and recomputed correlations in each bin. Interestingly, while we observed strong correlations for highly and moderately expressed genes, there was no significant correlation when restricting to lowly expressed genes (Fig. 4a, supplementary figs. S20 to S22, Supplementary Material online, supplementary table S2, Supplementary Material online). Next, we stratified genes based on the evolutionary constraint on the expression level or cell type-specificity of expression (using s_het_ [Zeng et al. 2024], a measure of the fitness effect of heterozygous loss of function, which typically corresponds to a ∼50% reduction in gene expression, and the τ metric [Yanai et al. 2005] respectively, supplementary tables S3 and S4, Supplementary Material online). While there was no difference in the correlation when stratifying by constraint on expression (supplementary figs. S23 to S25, Supplementary Material online, supplementary table S3, Supplementary Material online), we observed a much stronger negative correlation between the cell type proportion and the expression divergence for more cell type-specifically expressed genes (Fig. 4b, supplementary figs. S26 to S28, Supplementary Material online, supplementary table S4, Supplementary Material online). Since the expression level is also associated with cell type specificity, we tested whether these two properties were contributing independently to the negative correlations by stratifying genes by one of them while simultaneously controlling for the other. We found that both properties retained their predictive power even when controlling for the other (Fig. 4c and d, supplementary figs. S29 to S34, Supplementary Material online, supplementary tables S2 and S4, Supplementary Material online), suggesting independent contributions. We note that whether the weaker correlations we observed for lowly expressed genes were due to a true lack of association or simply less accurate expression level measurements remains an open question that will require larger datasets to explore. Overall, our results suggest that more highly expressed, cell type-specific genes are primarily driving the negative correlation between the cell type proportion and the gene expression divergence.

### Rapid Evolution of Layer 2/3 Intratelencephalic Neurons in the Human Lineage

Having identified this strong relationship between the cell type proportion and the evolutionary divergence, we reasoned that cell types with much faster divergence in the human lineage than expected based on their abundance may have been subject to atypical selective forces.

**Fig. 5.**
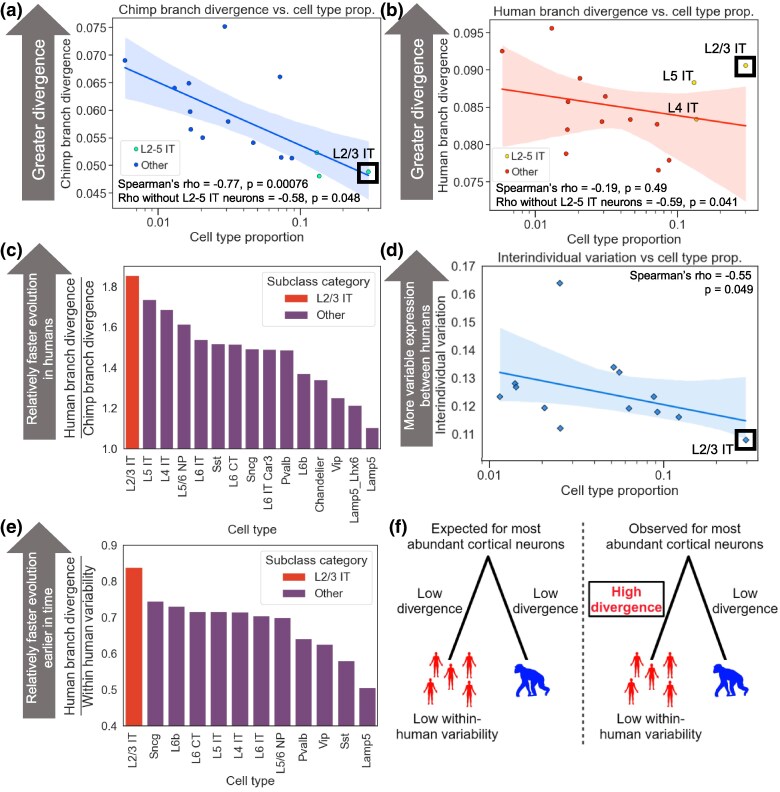
Accelerated evolution of L2/3 IT neurons in the human lineage. a) Plot showing the correlation between the neuronal subclass proportion (log_10_ scale on the *x*-axis) and the subclass-specific divergence on the chimpanzee branch in the MTG. Chimpanzee branch divergence was computed for each of 100 down-samplings, and the mean across those down-samplings is shown. The line and shaded region are the line of best fit from a linear regression and 95% confidence interval, respectively. The three rightmost points are L2-5 IT neurons. b) Same as in a) but for human branch divergence. The three rightmost points are L2-5 IT neurons. c) Bar plot showing the human branch divergence divided by the chimpanzee branch divergence for each subclass. d) Plot showing the correlation between the neuronal subclass proportion (log_10_ scale on the *x*-axis) and the subclass-specific interindividual variation across DLPFC samples from 25 human individuals. A representative iteration from 100 independent down-samplings is shown. Spearman's ρ and *P*-value shown are the median across 100 independent down-samplings (see Materials and Methods for details). The line and shaded region are the line of best fit from a linear regression and 95% confidence interval, respectively. e) Bar plot showing the human branch divergence divided by the within-human variability for each subclass. f) Conceptual model for accelerated evolution of L2/3 IT neurons in the human lineage. Made with BioRender.

To identify subclasses showing the most dramatic lineage-specific shifts in selection, we decomposed human–chimpanzee MTG expression divergence into its two components, divergence on the human branch and divergence on the chimpanzee branch. Applying the concept of parsimony—explaining the data with as few evolutionary transitions as possible—allows an outgroup species such as a gorilla to polarize changes and assign them to either the human or chimpanzee branch (see Materials and Methods). In the chimpanzee lineage, there was a strong negative correlation between the divergence and the subclass proportion (Fig. 5a, Spearman's ρ = −0.77, *P* = 0.00076), similar to the correlations between other primate species (Fig. 1a, supplementary fig. S1, Supplementary Material online). However, we observed a much weaker negative correlation in the human lineage (Fig. 5b, Spearman's ρ = −0.19, *P* = 0.49). The clearest outlier weakening the correlation was L2/3 IT neurons, the most abundant neuronal subclass, which diverged much faster than expected based on its proportion. This was also true to a lesser extent for the next two most abundant subclasses, L4 IT and L5 IT neurons. Indeed, removing these three subclasses substantially strengthened the negative correlation between the subclass proportion and the human-specific divergence (Fig. 5b; Spearman's ρ = −0.59, *P* = 0.041), making it indistinguishable from the corresponding chimpanzee-specific correlation (Fig. 5a; Spearman's ρ = −0.58, *P* = 0.048). Quantifying the magnitude of human acceleration (i.e. the rate of evolution in the human lineage relative to the chimpanzee lineage) for every subclass confirmed that L2/3 IT neurons underwent the greatest acceleration, followed by L4 and L5 IT neurons (Fig. 5c). We note that this is not at odds with the overall conservation of L2/3 IT neurons when compared to rhesus macaque or marmoset, as divergence along the human branch only represents a small fraction of the total divergence being measured in comparisons to monkeys.

An increased rate of evolution can involve either positive selection, favoring gene expression changes that increase fitness, or relaxed selective constraint in which random mutations are allowed to accumulate over time because they have little or no effect on fitness (Pollard et al. 2006). Although both positive selection and relaxed constraint can lead to similar patterns of lineage-specific acceleration, they imply very different underlying factors: positive selection is the force underlying nearly all evolutionary adaptation, while relaxed constraint is simply the weakening or absence of natural selection, which can lead to the passive deterioration of genes and their regulatory elements via mutation accumulation.

To distinguish whether positive selection or relaxed constraint was more likely to underlie the human-specific rapid evolution of IT neurons, we investigated the interindividual variability in expression of each neuronal subclass in the human population (Emani et al. 2024). If IT neurons evolved under reduced constraint in the human lineage, then we would expect them to have more variable expression among humans, leading to a weaker negative correlation between the subclass proportion and the interindividual variability. Instead, we observed a strong negative correlation between the subclass proportion and the interindividual variability in gene expression, with L2/3 IT neurons having the lowest variability of any subclass among humans (Fig. 5d, Spearman's ρ = −0.55, *P* = 0.049). Consistent with this, L2/3 IT neurons had the largest human branch divergence relative to their expression variability in modern humans (Fig. 5e). Overall, these results suggest that the rapid gene expression evolution of L2/3 IT neurons in the human lineage was unlikely to be due to relaxed constraint, and instead more likely the result of positive selection (Fig. 5f), though we cannot formally rule out other possible scenarios (see Discussion). In addition, it suggests that the relationship between the cell type proportion and the expression divergence holds within species as well as between species.

### Lower Expression of ASD-Linked Genes in Humans Compared to Chimpanzees

Our finding of human-specific accelerated evolution of L2/3 IT neurons raised the question of what phenotypes may be most affected by this. To explore this, we tested gene sets with strong evidence of linkage to specific human traits for bias toward higher or lower expression in humans relative to chimpanzees in L2/3 IT neurons. These gene sets were derived from two sources: the human phenotype ontology (HPO) (Köhler et al. 2021), a broad database covering hundreds of human traits, and SFARI, an ASD-specific database. Although ASD is often influenced by common genetic variants of small effect, which can be identified by Genome-wide association studies (GWAS), it can also be caused by single large-effect variants, typically causing loss-of-function of a core (Boyle et al. 2017) ASD gene. The SFARI database is the most comprehensive collection of these core genes (Abrahams et al. 2013 ); we refer to SFARI genes with a score of 1 as “high-confidence ASD-linked” and all SFARI genes, regardless of score, as “ASD-linked.” We restricted to only HPO gene sets with greater than 100 genes to ensure they were comparable with the SFARI gene set, which has 233 genes.

**Fig. 6.**
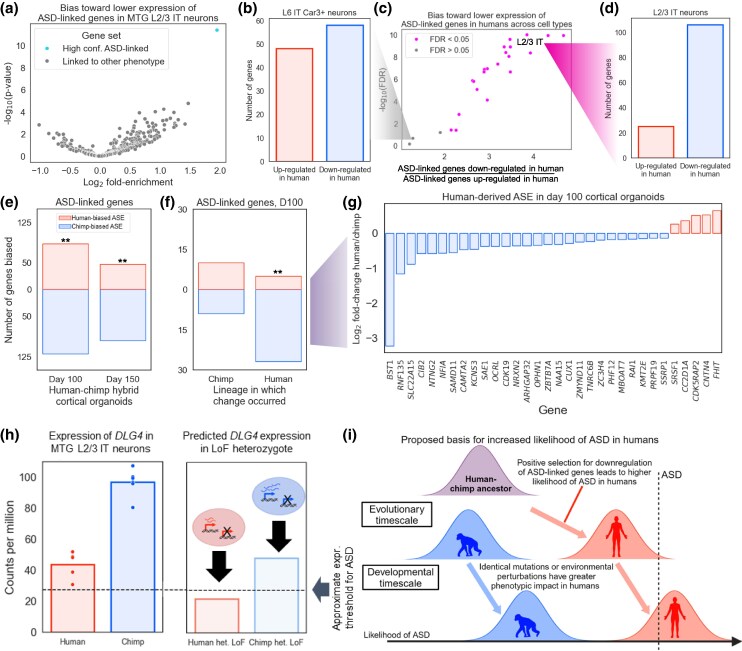
Positive selection for down-regulation of ASD-linked genes in the human lineage. a) Volcano plot showing the log_2_ fold enrichment for down-regulation in humans (*x*-axis) and the −log_10_ binomial *P*-value (*y*-axis). SFARI high-confidence ASD-linked genes are the rightmost point, all other categories of genes are from the Human Phenotype Ontology. Data are from MTG L2/3 IT neurons. b) Bar plot showing the number of high-confidence ASD-linked genes that are up-regulated vs. down-regulated in human relative to chimpanzee in the MTG L6 IT Car3+ neurons. c) Plot showing the fold enrichment for down-regulation in the human MTG (*x*-axis) and the −log_10_ binomial FDR (*y*-axis). Both neuronal and glial subclasses are included. Only subclasses with at least 500 human vs. chimpanzee differentially expressed genes in each direction are shown. d) Bar plot showing the number of high-confidence ASD-linked genes that are up-regulated vs. down-regulated in human relative to chimp in MTG L2/3 IT neurons. e) Bar plot showing the number of differentially expressed ASD-linked genes with higher allele-specific expression from the human allele and higher expression from the chimpanzee allele in cortical organoids. ** indicates binomial *P* < 0.01. f) Bar plot showing the number of differentially expressed ASD-linked genes with higher allele-specific expression from the human allele and higher expression from the chimpanzee allele in day 100 cortical organoids for human-derived and chimpanzee-derived genes separately. ** indicates binomial *P* < 0.01. g) Plot showing the log_2_ allele-specific expression ratios of differentially expressed, human-derived, ASD-linked genes in day 100 cortical organoids. Negative log_2_ fold-change indicates lower expression from the human allele. h) Left: Expression of *DLG4* in MTG L2/3 IT neurons; Right: Predicted expression of *DLG4* if one copy of the gene were non-functional. i) Conceptual model for how positive selection for down-regulation of ASD-linked genes led to a higher likelihood of ASD in humans compared to chimpanzees. Made with BioRender.

Strikingly, we found that high-confidence ASD-linked genes showed a stronger directionality bias in L2/3 IT neurons than any of the 359 HPO gene sets tested (4.0-fold enrichment for lower expression in the human MTG and 4.3-fold enrichment in the DLPFC; *P* < 10^−7^ for each; Fig. 6a, supplementary fig. S35a, Supplementary Material online). Although some HPO gene sets were also enriched, this was mostly a result of pleiotropic ASD-linked genes being present in multiple gene sets (supplementary fig. S35b and c, Supplementary Material online). This strong and specific enrichment for lower expression of high-confidence ASD-linked genes in human L2/3 IT neurons was intriguing, considering the known role of these neurons in ASD (Parikshak et al. 2013; Velmeshev et al. 2019; Pintacuda et al. 2023; Dear et al. 2024; Wamsley et al. 2024).

We then asked whether this lower expression of high-confidence ASD-linked genes was shared in other neuronal types beyond L2/3 IT. We found that some types of neurons had no significant directionality bias (Fig. 6b), while many subclasses shared a bias toward lower expression of high-confidence ASD-linked genes in humans compared to chimpanzees, suggesting that down-regulation of ASD-linked genes is broadly shared across neuronal types (Fig. 6c). In both the DLPFC and the MTG datasets, we observed the most significant trend toward lower human expression of these genes in L2/3 IT neurons (Fig. 6c and d, supplementary fig. S36a–c, Supplementary Material online).

Encouraged by these results, we tested whether genes linked to SCZ (Singh et al. 2022), another human-specific neuropsychiatric disorder, show a similar bias. We found an 8-fold enrichment for human down-regulation of SCZ-linked genes in the DLPFC L2/3 IT neurons (supplementary fig. S44a and b, Supplementary Material online). Although this is even stronger than the ASD bias, it only reaches a false discovery rate (FDR) < 0.05 in three MTG subclasses, such as Lamp5 and Pax6 inhibitory neurons, due to much lower statistical power (31 SCZ-linked genes vs. 233 high-confidence ASD-linked). Consistent with the known genetic overlap between ASD and SCZ, six of the SCZ-linked genes are also implicated in ASD, making it difficult to disentangle the signal from ASD and SCZ.

This excess of high-confidence ASD-linked genes with lower expression in humans is consistent with either down-regulation in the human lineage, up-regulation in the chimpanzee lineage, or a combination of both. To distinguish between these possibilities, we used the gorilla as an outgroup to assign each gene's expression divergence in the MTG to either the human or the chimpanzee lineage.

Comparing the expression of high-confidence ASD-linked genes in all three species revealed that the gorilla gene expression is significantly closer to chimpanzee, suggesting that there has been greater divergence in the human lineage (supplementary fig. S38a, Supplementary Material online). Consistent with this, a significantly larger number of high-confidence ASD-linked genes' expression diverged on the human branch than expected by chance in L2/3 IT neurons (supplementary fig. S38b, Supplementary Material online). In addition, human L2/3 IT neurons have overall lower expression of these genes as compared to all four NHPs in the dataset (supplementary fig. S38c, Supplementary Material online, supplementary table S5, Supplementary Material online). Overall, these results suggest a consistent pattern of human-specific down-regulation of ASD-associated genes in a neuronal cell type with a key role in ASD.

### Polygenic Positive Selection for Down-Regulation of ASD-Linked Genes in the Human Lineage

This human-specific down-regulation of high-confidence ASD-linked genes is striking and, based on the highly constrained expression of these genes, likely functionally significant. However, as with the accelerated evolution of L2/3 IT neurons discussed above (Fig. 5), the question of whether lineage-specific selection was responsible is key to understanding the factors that drove this divergence in the human lineage. Other potential explanations fall into two main categories. One is genetic changes that were not driven by selection, such as mutations that had little effect on fitness but became established in the human lineage through genetic drift. The other is non-genetic differences in the individuals sampled for these data sets; factors such as diet, environmental exposures, and age can impact the gene expression, but cannot be controlled in any comparison of tissue samples between humans and other species.

In order to definitively implicate lineage-specific selection, two steps are necessary. First, all non-genetic causes must be ruled out. Although this is not possible with tissue samples, it can be achieved *in vitro*. Human and chimpanzee induced pluripotent stem cells (iPSCs) can be fused to generate hybrid tetraploid iPSCs, which can then be differentiated into relevant cell types or organoids (Agoglia et al. 2021; Gokhman et al. 2021). In each hybrid cell, the human and chimpanzee genomes share precisely the same intracellular and extracellular environment. As a result, any difference in the relative expression levels of the human and chimpanzee alleles for the same gene—known as allele-specific expression (ASE)—reflects *cis*-regulatory changes between the two alleles. Both environmental and experimental sources of variability (including batch effects) are perfectly controlled in the hybrid system, since all comparisons are between alleles that share an identical environment and are present in the same experimental samples (Agoglia et al. 2021; Gokhman et al. 2021).

The second step necessary to infer lineage-specific selection is to test, and reject, a statistical “null model” of neutral evolution for the genetic component of divergence (Orr 1998). The simplest and most robust pattern predicted under neutral evolution of gene expression is the expectation that in a comparison between two species, genetic variants causing expression divergence will be just as likely to lead to higher expression in one species as in the other (Fraser 2011). For example, in a set of 20 functionally related genes, neutral evolution leads to a similar pattern as a series of 20 coin flips—an expectation of ∼10 genes more highly expressed in one species and ∼10 in the other, with deviation from this average following the binomial distribution (Fraser 2011). In contrast, natural selection that favors lower expression of these genes in one lineage will lead to a pattern of biased expression, with most of the 20 genes expressed at lower in that lineage (Fraser 2011). This framework, which has been applied extensively to gene expression and other quantitative traits (Orr 1998; Agoglia et al. 2021; Gokhman et al. 2021; Simon et al. 2024; Wang et al. 2024), is known as the sign test. Because the ASE of each gene in hybrid cells is generally independent of that of other genes (Fraser 2011), facilitating statistical analysis, hybrid ASE is ideally suited for detecting selection with the sign test, whereas the data from non-hybrids cannot be used in this manner. This statistical independence of each gene's ASE is a result of their independent *cis*-regulatory elements; since neighboring genes can sometimes share these elements, it is always advisable to confirm that the sign test results are not driven by groups of neighboring genes.

To apply this test for lineage-specific selection, we focused on a previously published RNA-seq dataset from human–chimpanzee hybrid cortical organoids (Agoglia et al. 2021). These organoids—which include glutamatergic and GABAergic neurons, astrocytes, and neural precursor cells—were sampled in a bulk RNA-seq time series of development *in vitro* (Agoglia et al. 2021 ); we focus on the two timepoints, day 100 and day 150, with the highest proportion of neurons. As described earlier, a significant bias in the directionality of ASE for any predefined set of genes can reject the null hypothesis of neutral evolution, and instead suggest lineage-specific selection. Applying this test to ASD-linked genes, we found a strong bias toward lower expression from the human allele in cortical organoids at two different stages of development (2.0-fold enrichment at day 100 of organoid development; binomial *P* = 0.003; Fig. 6e). The bias toward lower expression from human alleles was even stronger when using only high-confidence ASD-linked genes (2.5-fold enrichment; binomial *P* = 0.01 at day 100; supplementary fig. 39, Supplementary Material online). This ASE bias is inconsistent with neutral evolution and strongly implies the action of lineage-specific selection on the expression of ASD-linked genes.

In addition, although there are very few SCZ-linked genes with significant ASE in the hybrid cortical organoid data, among all SCZ-linked genes regardless of significance there is some bias toward human down-regulation, only reaching significance at day 150 (1.5-fold enrichment, binomial test *P* = 0.28 at day 100; 2.6-fold enrichment, binomial test *P* = 0.025 at day 150, supplementary fig. S40a and b, Supplementary Material online). We interpret these results as preliminary evidence that SCZ-linked genes may have also been subject to selection for down-regulation in the human lineage, though further work will be required to confirm this.

To determine the lineage (human or chimpanzee) on which the ASD-linked gene expression changes occurred, for genes with matching directionality in the L2/3 IT and organoid data, we once again polarized gene expression divergence in the MTG into human-derived and chimpanzee-derived categories using gorilla as an outgroup. Out of 17 chimpanzee-derived genes, there was no directionality bias in the organoid ASE data at either day 100 or day 150 (9 out of 19 with lower expression from the human allele at day 100, Fig. 6f and g, supplementary fig. 41, Supplementary Material online), consistent with neutral evolution. However, out of 32 human-derived genes, 27 had lower expression from the human allele (Fisher's exact test *P* = 0.010 at day 100, odds ratio = 6.0; *P* = 0.010, odds ratio = 8.9 at day 150; Fig. 6f and g, supplementary fig. 41, Supplementary Material online). This trend is even stronger when using a more relaxed FDR cutoff of 0.1 (34 down-regulated in human vs. 5 up-regulated; Fisher's exact test *P* = 0.0043, odds ratio = 5.9; *P* = 0.0017, odds ratio = 12.5 at day 150). Overall, this strongly suggests that many ASD-linked genes were down-regulated specifically in the human lineage.

This coordinated down-regulation of 34 ASD-linked genes could conceivably be due to either positive selection or loss of constraint, as both of these types of lineage-specific selection could lead to down-regulation (Fraser 2011; Simon et al. 2024). To determine if ASD-linked genes might be evolving under relaxed constraint in humans, we tested several predictions of the relaxed constraint model. First, genes evolving under relaxed constraint might be expected to have accumulated more substitutions affecting protein sequence and/or gene expression in the human lineage. However, we found no difference in the protein sequence constraint (measured by dN/dS [Gayà-Vidal and Albà 2014]) or the number of mutations near the transcription start site (TSS) between humans and chimpanzees (after correcting for genome-wide differences between the two lineages, *P* = 0.42 for dN/dS, *P* = 0.24 for mutations near TSS, paired *t*-test, supplementary fig. 42a and b, Supplementary Material online). In addition, the expression of genes evolving under relaxed constraint in humans would likely be more variable across human individuals compared to chimpanzee individuals. However, we found the opposite for ASD-linked genes—slightly less variability in expression in humans (*P* = 0.08 for the DLPFC, *P* = 2.5×10^−5^ for the MTG, paired *t*-test, supplementary fig. 42c and d, Supplementary material online), suggesting that the expression of ASD-linked genes may actually be under stronger constraint in humans compared to chimpanzees. Consistent with this, the vast majority of ASD-linked genes have strongly constrained expression in humans as measured by loss-of-function intolerance (82% of ASD-linked genes have probability of loss-of-function intolerance [Chen et al. 2024] >0.9 compared to 17% genome-wide and 24% of genes expressed in L2/3 IT neurons; similarly, 82% of ASD-linked genes have a fitness effect of heterozygous loss of function [Zeng et al. 2024] [s_het_] > 0.1, compared to 18% genome-wide and 24% of genes expressed in L2/3 IT neurons).

Next, we explored whether particular subsets of ASD-linked genes had a stronger bias toward down-regulation than other ASD-linked genes. ASD-linked genes tend to encode proteins that localize to the synapse, encode transcription factors (TFs) or chromatin remodelers (CRs), and/or be haploinsufficient (Satterstrom et al. 2020). When splitting ASD-linked genes into these three partially overlapping categories, we found comparable human down-regulation in all groups (supplementary fig. 43, Supplementary Material online). For example, 83% of ASD-linked haploinsufficient genes were down-regulated, which is similar to the 75% of ASD-linked non-haploinsufficient genes that were down-regulated (supplementary fig. S43a, Supplementary Material online). This suggests that ASD-linked genes in general, rather than one of these specific subcategories, are biased toward down-regulation. Finally, we tested whether synaptic genes, TFs/CRs, or haploinsufficient genes in general tend to be down-regulated in the human lineage. We found that all three categories tend to have lower expression in humans compared to chimpanzee L2/3 IT neurons (supplementary fig. 44, Supplementary Material online). Overall, this suggests that the down-regulation of ASD-linked genes we observed may be part of a larger trend extending to other genes with similar properties as ASD-linked genes, consistent with previous work on human-specific synaptic gene expression (Jorstad et al. 2023).

Although we cannot rule out any possibility of relaxed constraint at some point in the past, these results favor a model in which polygenic positive selection acted to decrease expression of ASD-linked genes in some types of human neocortical neurons, including L2/3 IT neurons (Fig. 6b). As a ∼50% reduction in the gene expression underlies increased probability of the ASD diagnosis for the vast majority of these genes (Satterstrom et al. 2020), this suggests that down-regulation of ASD-linked genes may have increased ASD prevalence by bringing humans closer to a hypothetical “ASD expression threshold” below which ASD characteristics manifest. As an example, *DLG4*, which encodes the key synaptic protein PSD-95 and for which loss of one copy causes ASD (Rodríguez-Palmero et al. 2021), has 2.5-fold lower expression in humans compared to chimpanzees (Fig. 6h). Consistent with this, it also has 2.5-fold lower protein abundance in the postsynaptic density in humans compared to rhesus macaques, and 3.4-fold lower protein abundance in humans compared to mice (Wang et al. 2023) (human vs. rhesus *t*-test *P* = 0.0028, human vs. mouse *t*-test *P* = 0.00014, supplementary fig. 45, Supplementary Material online). While this human-specific down-regulation (supplementary table S5, Supplementary Material online) that led to the current human baseline expression level of *DLG4* is not sufficient to cause ASD, further down-regulation via loss of a single copy may push humans below the ASD expression threshold whereas loss of a single copy in chimpanzees would maintain expression above this threshold (Fig. 6h). Although these genes are linked to ASD primarily due to their monogenic effects, the majority of ASD cases are thought to be caused by many small genetic and environmental perturbations collectively pushing individuals past some threshold (Autism Spectrum Disorder Working Group of the Psychiatric Genomics Consortium et al. 2019). We propose that the down-regulation of ASD-linked genes in humans increased the likelihood of ASD in the human lineage, such that small perturbations during development are sufficient to cause ASD characteristics in humans but not chimpanzees (Fig. 6i).

## Discussion

Building on an analogy between genes and cell types, we have identified a general principle underlying the rate of evolution of different neuronal types in the mammalian neocortex. We found a strong negative correlation between the abundance of each neuronal cell type and the rate at which its gene expression levels diverge across six mammalian species and three independent datasets (Bakken et al. 2021a; Ma et al. 2022; Jorstad et al. 2023). Interestingly, this correlation remained very strong when collectively analyzing inhibitory and excitatory neurons, despite their very different developmental origins and functions (Molyneaux et al. 2007; Lim et al. 2018).

Based on this initial discovery, we found that L2/3 IT neurons evolved unexpectedly quickly in the human lineage compared to other apes. This accelerated evolution included the disproportionate down-regulation of genes associated with autism spectrum disorder and schizophrenia, two neurological disorders closely linked to L2/3 IT neurons that are common in humans but rare in other apes. Finally, we found that this down-regulation, present both in adult neurons and in organoid models of the developing brain, was likely due to polygenic positive selection on *cis*-regulation. These results differ from, but do not contradict, previous findings that a group of synapse genes shows human-specific up-regulation during early development that is disrupted in people with ASD (Liu et al. 2016). Interestingly, it was recently proposed that lower expression of ASD-linked genes in males than females may magnify the effects of haploinsufficiency of these genes in males, leading to a higher risk for ASD in males (Velmeshev et al. 2023). This mirrors our proposal that lower expression of ASD-linked genes in humans than in chimpanzees increases risk for ASD in the human lineage. Overall, our analysis suggests that natural selection on gene expression may have increased the prevalence of ASD, and perhaps also SCZ, in humans (Fig. 6h).

Although it has been widely hypothesized that natural selection for human-specific traits has increased human disease risk (Crow 1997; Varki 2001; Vasseur and Quintana-Murci 2013; Benton et al. 2021; Zug and Uller 2022), unambiguous evidence for this has been lacking. While there is strong evidence linking natural selection on within-human genetic variation to disease risk (e.g. sickle cell disease [Sabeti et al. 2006]), it has proven far more challenging to find similar examples involving genetic variants shared by all humans. There are human–chimpanzee differences that have been linked to interspecies differences in disease risk (e.g. human-specific pseudogenization of the *CMAH* gene, which is thought to have shaped human susceptibility to infectious diseases [Chou et al. 1998; Varki 2001; Dankwa et al. 2016]), but there is no evidence for positive selection on these interspecies genetic differences. In addition, while there are many examples of positive selection on human–chimpanzee differences (Enard et al. 2014; Gayà-Vidal and Albà 2014; Agoglia et al. 2021; Gokhman et al. 2021; Starr et al. 2023; Wang et al. 2024), these changes have no clear link to the likelihood of diseases or disorders in humans. Finally, although the enrichment for ASD-linked variants within HARs (Doan et al. 2016; Shin et al. 2024) is suggestive of a role for human–chimpanzee differences in HARs (many of which are thought to be positively selected [Pollard et al. 2006]) in increasing the likelihood of ASD in humans, a connection between those human–chimpanzee differences and ASD has not been established. Overall, our findings provide the strongest evidence to date supporting the long-standing hypothesis that natural selection for human-specific traits has increased the likelihood of certain disorders.

Although our results strongly suggest natural selection for down-regulation of ASD-linked genes, the reason why this conferred fitness benefits to our ancestors remains an open question. Answering this question is difficult in part because we do not know what human-specific features of cognition, brain anatomy, and neuronal wiring gave our ancestors a fitness advantage, but we can speculate about two general classes of evolutionary scenarios. First, down-regulation of ASD-linked genes may have led to uniquely human phenotypes. For example, haploinsufficiency of many ASD-linked genes is associated with developmental delay (Zug and Uller 2022), so their down-regulation could have contributed to the slower postnatal brain development in humans compared to chimpanzees. Alternatively, capacity for speech production and comprehension is unique to or greatly expanded in humans and often impacted in ASD and SCZ (Chang et al. 2022; Vogindroukas et al. 2022). If down-regulation of ASD-linked genes conferred a fitness advantage by slowing postnatal brain development or increasing the capacity for language, that could result in the signal of positive selection we observed.

On the other hand, the down-regulation we observed may have been compensatory and reduced the negative effects of some other human-specific trait or traits. For example, the ratio of excitatory and inhibitory synapses on pyramidal neurons is fairly constant between humans and rodents despite massive differences in brain and neuron size (DeFelipe et al. 2002). In addition, excitatory-inhibitory imbalance is a leading hypothesis for the circuit basis of ASD (Sohal and Rubenstein 2019). If human brain expansion, changes in metabolism, or any other factor shifted this balance away from the fitness optimum, down-regulation of ASD-linked genes could potentially compensate. Overall, more work is needed to understand how natural selection acting on the expression of ASD-linked genes in the human lineage may have shaped human phenotypes.

Our results come with important caveats. As with most correlations, causality is not implied. Our initial hypothesis was that cell type proportions may affect evolutionary rates via more severe fitness effects of expression changes in more abundant cell types, leading to greater evolutionary constraint than in rare cell types (Fig. 1a). While this is a plausible explanation for our results, there also may be unknown correlates of cell type proportion that are causal. We leave explicit testing of this model to future work. Another caveat is that the cortical organoid data are from a different developmental timepoint and mix of cell types than the pure population of adult L2/3 IT neurons we focus on. In the future, it will be informative to investigate more mature populations of human–chimp hybrid excitatory neurons *in vitro* to compare with adult L2/3 IT neurons *in vivo*. As with most studies of non-human great apes, the sample sizes used here (ranging from 4 to 7 individuals) are relatively small, and it will be important to confirm these results with larger cohorts. This will also enable more thorough investigation of lowly expressed genes. Finally, although changes in subtype proportions can give rise to apparent accelerated evolution, the L2/3 acceleration we observed is broadly distributed across L2/3 subtypes (not shown), suggesting that changes in relative subtype abundances do not explain this result.

Along with establishing a mechanism underlying these correlations, another exciting future direction will be to explore this phenomenon in other tissues and brain regions. While cross-species atlases from other brain regions exist, they generally lack a sufficient number of cells profiled (Bakken et al. 2021b; Kamath et al. 2022 ) or fail to meet our inclusion criteria in other ways (see Materials and Methods). However, this will become increasingly feasible as additional large cross-species snRNA-seq studies are published and enable exploration of how generalizable our results are across tissues. An especially interesting question will be whether rare but vital neuron cell types (e.g. serotonergic or dopaminergic neurons [Weiger 1997; Bromberg-Martin et al. 2010]) follow the same pattern we have observed for neocortical neurons; this will help distinguish between cell type abundance vs. importance as the driving factor underlying the relationship we have observed. It will also be interesting to explore what factors are associated with the rate of cell type-specific gene expression divergence in contexts that lack stable cell type proportions (e.g. during development or in the immune system). Finally, it is worth noting that the cell type proportion is unlikely to be the sole driver of differences in evolutionary rate between cell types. For example, oligodendrocytes and astrocytes are more common than neurons in some parts of the brain, yet evolve more quickly than neurons, hinting at additional core principles governing the rate at which cell types evolve (Pembroke et al. 2021). We leave exploration of this question to future work.

Considering that many ASD-linked genes are extremely sensitive to perturbations in their expression, our findings raise the important question of how significant reductions in the expression of so many dosage-sensitive genes were tolerated in the human lineage. As haploinsufficiency of many of these genes has severe fitness consequences in both humans and mice (Zug and Uller 2022), it is unlikely that these changes occurred through single mutations of large effect. In addition, our analysis of allele-specific expression suggests that *cis*-regulatory changes underlie many of the gene expression changes we observe. Therefore, we favor a model in which many *cis*-acting mutations of small effect are fixed over time, eventually leading to the large-scale down-regulation of ASD-linked genes in the human lineage. It will be interesting to use deep learning predictions of variant effects combined with experimental validation to identify the genetic differences underlying changes in the expression of ASD-linked genes in the human lineage.

It is also possible that the down-regulation of many ASD-linked genes is less deleterious than the down-regulation of a single gene. As an analogy, whole-genome duplications can be well-tolerated in vertebrates, even though duplication of some individual genes—including many of those linked to ASD—can be far more deleterious. An intuitive explanation for this counterintuitive observation is that relative expression levels, or stoichiometry, could impact fitness even more than absolute expression levels (Darnell 2020). Under this model, the key idea is that the down-regulation of many ASD-linked genes would have less impact on their relative levels than a change in the expression of a single gene. Finally, it is worth considering an alternative model. Rather than pushing humans closer to some gene expression threshold beyond which ASD-like traits manifest, down-regulation of ASD-linked genes may have made neuronal networks more sensitive to genetic or environmental perturbations. In this model, humans are no closer to the ASD-like trait threshold in the absence of some perturbations, but identical perturbations would have larger effects in humans and be more likely to result in ASD-like traits. Excitingly, Clustered interspaced short palindromic repeats (CRISPR) or CRISPR-based methods to precisely manipulate the expression levels of many genes at once may soon allow us to more directly test these hypotheses. Overall, it will be important to develop a deeper understanding of how cell types and genes implicated in ASD and SCZ have evolved in the human lineage, as this will improve our understanding of uniquely human traits and neuropsychiatric disorders.

## Materials and Methods

### Quantifying Cell Type-Specific Gene Expression Divergence Between Species

We analyzed three main datasets in this study, which we refer to by the cortical area sampled (MTG, DLPFC, M1). These were the only studies meeting both of our inclusion criteria: multiple species profiled in the same study using the same snRNA-seq protocols for each species within a study, and at least 10 orthologous cell types having 250 or more cells per species. The following are examples of studies that did not meet these inclusion criteria:

- A multi-species study of the retina used different protocols for different species, and not all species were sampled as part of the same original study. For example, different antibodies were used to enrich for subpopulations of cells in different species, and some species did not have a sufficient number of cells profiled without enrichment to accurately estimate cell type proportions (Hahn et al. 2023).
- A multi-species study of substantia nigra dopaminergic neurons did not have a sufficient number of cells profiled per species (Kamath et al. 2022).
- A multi-species study of the lateral geniculate nucleus did not profile enough cells per species, and their dissection scheme was incompatible with estimating neuronal cell type proportions (Bakken et al. 2021b).

All statistical tests and analyses were performed in Python using scipy v1.10.1 (Virtanen et al. 2020) except for the DESeq2 analysis. For the M1 and MTG data, we converted from RDS files to h5 files using Seurat and Seurat Disk (Hao et al. 2021). We conducted all analyses within each dataset to avoid batch effects from comparing across datasets. We used the cell type annotations and count matrices directly from the study that first reported the dataset in conjunction with scanpy v1.7.2 (Wolf et al. 2018). The procedure outlined below was performed 100 times independently on each dataset unless otherwise noted. To quantify cell type-specific expression divergence without confounding with cell type proportion, we first down-sampled the number of cells in each cell type so that it was equal across all cell types and species. We down-sampled without replacement to 250 cells at the subclass level and 50 cells at the subtype level for the main analysis presented in the text. Only subclasses and subtypes with at least this many cells were included in downstream analysis. We then restricted to five-way one-to-one protein-coding non-mitochondrial orthologs (downloaded from Ensembl Biomart for hg38) (Yates et al. 2022) between human, chimpanzee, gorilla, rhesus macaque, and marmoset for the MTG and DLPFC data, and three-way one-to-one orthologs for human, marmoset, and mouse for the M1 dataset. We then summed the expression across all cells within a cell type to create a pseudobulked expression profile for that cell type.

For each possible pairwise comparison between species, we down-sampled the total counts in each cell type so that it was equal across all cell types for both species in the comparison. We then computed counts per million (CPM) in each cell type. After computing CPM, we filtered out genes with (1) fewer than 25 counts in both species or (2) fewer than 1 CPM in both species per cell type. As a result, if a gene passed the filtering criteria in one cell type but not another, it would be included only for the cell type in which it passed the filtering criteria. We then computed the log_2_(CPM) and used the Spearman correlation distance, which is nonparametric and robust to outliers, to measure the gene expression divergence between species in each cell type. We focused on the Spearman correlation and not more sophisticated models (e.g. generalized linear mixed models) for two reasons beyond those outlined above. First, non-ape samples were generally sex-balanced, had similar ages, and had very low postmortem intervals, suggesting very minimal sources of variation outside of species. Second, principal component analysis revealed that the first principal component corresponded to species and explained a large majority of the variance. For example, and consistent with the first point, PC1 corresponded to species and explained 88% of the variance when comparing macaque and marmoset DLPFC L2/3 IT neurons. Similarly, for human and chimpanzee, PC1 corresponded to species and explained 87% of the variance (PC2 explained 4% of the variance). Overall, this suggests that any potentially confounding sources of variation are minimal, supporting the use of the Spearman correlation.

Notably, this process involved several analysis decisions that could affect our results. To test how robust our results were to these choices, we tested all combinations of the following:

1. Down-sampling to 50, 100, 250, or 500 cells.
2. Filtering genes with fewer than 5, 10, 25, or 50 counts.
3. Filtering genes with fewer than 1 or 5 CPM.
4. Using log_2_(CPM) or not log transforming.
5. Using the Spearman correlation distance, Pearson correlation distance, Euclidean distance, or L1 distance metrics.

In general, our results were robust to any combination of these parameters (supplementary tables, Supplementary Material online). When stratifying, we only used a subset of these combinations due to the greater number of computations required. Although we considered applying a variance stabilizing transformation (e.g. the log of raw expression plus a pseudocount) to the single-cell data before pseudobulking, we think that the chosen methodology is most consistent with standard practices in the field (Squair et al. 2021) and likely best reflects the expression level of genes had we performed bulk RNA-seq on pure populations of cells. However, we verified that our results were robust to applying a variance stabilizing transformation. For example, applying the log(read count plus 1) transformation (Ahlmann-Eltze and Huber 2023) to all neurons in the DLPFC at the subclass level, this results in Spearman's ρ equal to −0.68 (*P* = 0.0029), which is quite similar to the value of −0.76 shown in Fig. 1c.

### Computing Cell Type Proportions and Correlation With Gene Expression Divergence

All three datasets were generated with snRNA-seq and so likely accurately represent the true proportion of neuronal cell types in the neocortex (Ding et al. 2020). To compute cell type proportions, we restricted to neuronal cells with greater than or equal to the number of cells we down-sampled to. We then computed cell type proportion separately for each species by dividing the number of cells of each type by the total number of cells profiled. For each interspecies comparison, we averaged the cell type proportion across both species. We then computed the Spearman correlation between the averaged cell type proportions and the cell type-specific gene expression divergence computed as described above. As we did this across 100 independent down-samplings (numbered 1 to 100), we reported the median Spearman's ρ and *P*-value throughout the text and figures. If there was an individual down-sampling iteration that had the median Spearman's ρ and *P*-value, we made the scatterplots shown in Figs. 1 to 4 using the first such iteration. If no iteration had the median ρ and *P*-value, we showed the iteration closest to the median with the greatest number of iterations that had ρ and *P*-value. For example, if 22 iterations resulted in ρ = −0.5 and 19 iterations resulted in ρ = −0.6, both of which were closest to the median of −0.55, then an iteration with −0.5 would be shown. If there was still a tie after this process, we showed the iteration with the lowest number. Because the Spearman correlation is a nonparametric rank-based test, it is unaffected by any rank-preserving transformation of the data; therefore, our choice to show scatter plots with log-transformed cell type proportions was for visualization only and had no effect on the results.

To estimate divergence along the human branch, we used the formula


$$\frac{\mathrm{HC}\;\mathrm{divergence}+\mathrm{HG}\;\mathrm{divergence}-\mathrm{CG}\;\mathrm{divergence}}{2}$$

Here HC stands for human–chimp, HG stands for human–gorilla, and CG stands for chimp–gorilla.

Similarly, to estimate divergence along the chimp branch, we used the formula


$$\frac{\mathrm{HC}\;\mathrm{divergence}+\mathrm{CG}\;\mathrm{divergence}-\mathrm{HG}\;\mathrm{divergence}}{2}$$

### Stratifying by Expression Level, Cell Type-Specificity of Expression, and Constraint on Expression

To stratify by expression level, we ranked genes by the average CPM between the two species being compared for each cell type separately. We then assigned the top third of genes with the highest expression to the highly expressed bin, the next third to the moderately expressed bin, and the remaining third to the lowly expressed bin. Whenever we stratified by expression level or another metric, we used the Euclidean distance to measure the gene expression divergence because the limited dynamic range of expression for the moderately and lowly expressed bins led to unrealistically high correlation distances. Similarly, we ranked genes by τ (Yanai et al. 2005), a measure of how cell type-specifically a gene is expressed, and split those genes into three bins. We computed τ separately for both species across all subclasses or subtypes with a sufficient number of cells and then computed the average value for each gene. For constraint on expression, we considered all genes with heterozygous loss of function (corresponding to an approximately 50% reduction in the gene expression) fitness effect (Zeng et al. 2024) s_het_ > 0.1 to be highly constrained, genes with s_het_ between 0.1 and 0.01 as moderately constrained, and the remaining genes with s_het_ < 0.01 to be lowly constrained. Because there was a different number of genes in each bin in this case, we down-sampled genes to reach an equal number in each bin.

When controlling for expression level and stratifying by τ, we compared the high bin with the moderate and low bins separately. To control expression, we first computed the log_2_ fold-change between all genes in the high bin and all genes in the moderate or low bin, and restricted to pairs of genes with absolute log_2_ fold-change less than 0.05. We then split this list of gene pairs into those with a negative log_2_ fold-change, positive log_2_ fold-change, and zero log_2_ fold-change, shuffled the list, and removed duplicate genes. We kept all gene pairs with a log_2_ fold-change of zero and down-sampled the list of gene pairs with positive or negative log_2_ fold-change so that there were an equal number in each category. This resulted in a final set of genes in the high bin with matched expression to genes in the moderate or low bin, which we used to compute cell type-specific gene expression divergence. When controlling for τ, we applied the same strategy but required an absolute log_2_ fold-change less than 0.01.

### Comparing Interindividual Variability in Gene Expression and Cell Type Proportion

To measure the within-human interindividual variation in cell type-specific gene expression, we used a uniformly processed dataset from the DLPFC (Emani et al. 2024). We restricted to control samples from individuals of European ancestry with an age of death greater than or equal to 25. We selected 13 neuronal subclasses for which the majority of individuals had greater than 50 nuclei profiled for further analysis, and restricted to samples with greater than or equal to 50 nuclei for all 13 subclasses. After this filtering process, 25 samples remained. Next, we down-sampled to 50 nuclei from each subclass in each dataset and computed pseudobulked counts. We then down-sampled counts so that there was an equal number of total counts across all subclasses for each individual. For each subclass, we removed genes with average counts across all individuals less than 25 and computed CPM. We then computed the Spearman correlation distance between each sample and the mean expression profile across all samples and took the mean of those 25 correlation distances as our measure of cell type-specific gene expression variation within humans. We performed this procedure across 100 independent down-samplings. To estimate cell type proportions, we computed the cell type proportions for the 13 subclasses and averaged them together. We then computed the Spearman correlation between the subclass-specific interindividual variation and the cell type proportions across the 100 down-samplings. We report the median Spearman's ρ and *P*-value across the 100 down-samplings and show the first down-sampling with the median Spearman's ρ and *P*-value in Fig. 5d.

### Analysis of ASD- and SCZ-Linked Genes in snRNA-seq Data

We used the SFARI gene database of ASD-linked genes and considered any genes with a score of 1 to be “high-confidence” (233 total) and all genes, regardless of score to be all ASD-linked genes (1,176 genes) (Abrahams et al. 2013). As we are not aware of a similar resource for SCZ, we used the 31 genes with FDR < 0.1 in a recent rare variant association study for SCZ (Singh et al. 2022). We do not use GWAS in this analysis as it is often difficult to determine the true causal gene(s) and their direction of effect (e.g. whether increased or decreased gene expression is associated with the disorder). Although this limits the number of traits we can test, it ensures that the gene-trait links we analyze are causal and have large effects with known directionality. Throughout, FDRs were corrected for multiple tests with the Benajmini–Hochberg method. To identify differentially expressed (DE) genes and compute log_2_ fold-changes between species, we ran DESeq2 (Love et al. 2014) on the subclass-level pseudobulked counts and used apeglm (Zhu et al. 2019) to shrink the log_2_ fold-changes. To test for a bias toward lower expression of ASD- and SCZ-linked genes in each cell type, we restricted to genes with FDR < 0.05 in the human–chimpanzee comparison and used the binomial test comparing the number of genes with negative log_2_ fold-change (i.e. higher expression in chimpanzee) to the number of genes with positive log_2_ fold-change. We used the frequency of negative log_2_ fold-changes among all genes with FDR < 0.05 as the background probability in the binomial test. We repeated this for both high-confidence and all ASD-linked genes.

To determine whether the higher expression in chimpanzees relative to humans was more likely due to changes on the chimpanzee branch or the human branch, we first filtered to only high-confidence ASD-linked genes that were differentially expressed between chimpanzees and gorillas in L2/3 IT neurons. Genes were assigned as having a significant human-derived or chimpanzee-derived expression change in the MTG dataset by comparison with the human–gorilla and chimpanzee–gorilla log_2_ fold-changes. First, if the absolute human–gorilla and chimpanzee–gorilla log_2_ fold-change were both greater than the absolute human–chimpanzee log_2_ fold-change, that gene was considered ambiguous. After removing ambiguous genes, a gene was considered as having a human-derived expression change if the absolute human–gorilla log_2_ fold-change was greater than the absolute human–chimpanzee log_2_ fold-change and vice versa for chimpanzee-derived. To generate supplementary table S5, Supplementary Material online, we used strict criteria to call genes as having a human-specific gene expression change in the MTG data, requiring that a gene be differentially expressed (i.e. FDR < 0.05) for each human-NHP comparison with the same direction of differential expression. We then added the SFARI score and whether a gene is considered syndromic, and only included genes that are differentially expressed (FDR < 0.05) between the human and the chimpanzee.

### Analysis of ASD-Linked Genes in Human–Chimpanzee Hybrid Cortical Organoid Data

We used the previously described dataset from human–chimpanzee cortical organoids, reprocessed as previously described (Starr et al. 2023). Briefly, reads were aligned to the human (hg38) and chimpanzee (PanTro6) genomes with STAR and corrected for mapping bias using Hornet (Van De Geijn et al. 2015). Reads were assigned to the human or chimpanzee allele using a set of high-confidence human–chimp single nucleotide differences and collapsed to counts per gene with ASEr. DESeq2 (Love et al. 2014) was used to identify genes with significant ASE with the hybrid line that each sample was from used as a covariate. DESeq2 (Love et al. 2014) and apeglm (Zhu et al. 2019) were used to compute log_2_ fold-changes. For the below analyses, we used the chimpanzee-aligned data, which has a very slight bias toward higher expression from the human allele. It has previously been shown that tetraploidy has little to no effect on gene expression in these cells (Song et al. 2021).

To test for a significant bias toward down- or up-regulation from the human allele for ASD- or SCZ-linked genes, we restricted to genes with FDR < 0.05 in the cortical organoid data and intersected those genes with the list of ASD- or SCZ-linked genes. We then used the binomial test comparing the number of genes with negative log_2_ fold-change (i.e. higher expression in the chimpanzee) to the number of genes with positive log_2_ fold-change. We used the frequency of negative log_2_ fold-changes among all genes with FDR < 0.05 as the background probability in the binomial test. We repeated this for both high-confidence and all ASD-linked genes. To investigate whether these *cis*-regulatory changes likely occurred in the human or chimpanzee lineage, we used the assignments as human- or chimpanzee-derived from L2/3 IT neurons in the MTG dataset described above. For genes that had matching human–chimpanzee log_2_ fold-change sign in both the MTG and cortical organoid datasets, we created a 2×2 table of human/chimp-derived and down/up-regulated from the human allele and applied Fisher's exact test.

### Analysis of Constraint on ASD-Linked Genes in Humans and Chimpanzees

We used previously published dN/dS estimates (Gayà-Vidal and Albà 2014) and restricted only to genes with at least one synonymous and nonsynonymous difference on both the human and chimpanzee branches. We compared dN/dS values for ASD-linked genes with a paired *t*-test. To compute the number of genetic differences within 5 kb of the TSS for each lineage, we used our previously described set of high-confidence human–chimpanzee single nucleotide genetic differences (Starr et al. 2023). Briefly, this was created by identifying all single nucleotide differences between PanTro6 and hg38 and filtering out sites that were not homozygous for the reference allele in three humans and three chimpanzees. We then intersected this with a previously described list of human–chimpanzee orthologous TSS expanded by 2.5 kb on either side and restricted to only TSS for ASD-linked genes (Wang et al. 2024). To correct for the slightly larger number of human-derived sites across all genes, we down-sampled the human-derived variants near the TSS of ASD-linked genes, keeping a fraction of sites equal to the total number of chimp-derived genetic differences divided by the total number of human-derived genetic differences. We then used a paired *t*-test to compare the two distributions.

To compare the within-species variance for humans and chimpanzees in expression of ASD-linked genes, we computed the variance in pseudobulked CPM from L2/3 IT neurons across individuals in the DLPFC and MTG separately. As the mean expression level and batch effects can have a major impact on the expression variance, we normalized the variance to the variance of the 100 genes with the closest mean expression to each ASD-linked gene. To do this, we computed the fraction of those 100 genes with smaller variance than the focal ASD-linked gene in each species and dataset separately. We then compared the values in humans and chimpanzees with a paired *t*-test.

### Comparing Different Phenotypes and Gene Categories to ASD-Linked Genes

To compare down-regulation of high-confidence ASD-linked genes to genes associated with other phenotypes, we used the HPO, restricting to phenotypes with at least 100 genes. We tested all these gene sets in addition to the high-confidence ASD-linked genes and computed fold-enrichment as described above for ASD-linked genes. We controlled for gene expression as described in the “Stratifying by expression level, cell type-specificity of expression, and constraint on expression” section, filtering out all gene pairs with an absolute log fold-change greater than 0.1.

To subset ASD-linked genes, we used all genes present in the SynGo database (Koopmans et al. 2019) as our list of synaptic genes, all genes classified as “1—Monomer or homomultimer,” “2—Obligate heteromer,” “3—Low specificity DNA-binding protein” from Lambert et al. (2018) as our list of transcription factors and chromatin remodelers, and all genes with pLI > 0.9 from gnomad version 4.1 (Chen et al. 2024) as our list of haploinsufficient genes. We intersected these with the set of ASD-linked genes with these lists and removed all ASD-linked genes from those lists to define genes as “ASD-linked and in a category” or “ASD-linked and not in a category,” respectively. We also removed genes in a category from the list of ASD-linked genes to define the list of genes that are ASD-linked and, for example, not synaptic. When working with the MTG data, we always subsequently restricted to high-confidence ASD-linked genes. With these categories in hand, we then computed the proportion of genes in each category that are down-regulated. We used the binomtest function from scipy with the background probability set to the proportion of genes in a category not linked to ASD that are down-regulated to test whether ASD-linked genes within a particular category were more down-regulated than genes in the category that are not linked to ASD.

### Analysis of Postsynaptic Proteomics Data

We plotted PSD-95 protein abundances from the supplemental materials of Wang et al. (2023). We used the *t*-test to compare levels between species.

## Supplementary Material

msaf189_Supplementary_Data

## Data Availability

The MTG data are available from https://labshare.cshl.edu/shares/gillislab/resource/Primate_MTG_coexp/Great_Ape_Data/. The metadata for the MTG study are available from https://github.com/AllenInstitute/Great_Ape_MTG/blob/master/data/ (files ending in “for_plots_and_sharing_12_16_21.RDS”). The DLPFC data are available from https://data.nemoarchive.org/biccn/grant/u01_sestan/sestan/transcriptome/sncell/10x_v3/. The M1 data are available from https://data.nemoarchive.org/publication_release/Lein_2020_M1_study_analysis/Transcriptomics/sncell/10X/. The constraint metric s_het_ was downloaded from the supplemental materials of https://www.biorxiv.org/content/10.1101/2023.05.19.541520v1. The constraint metric pLI was downloaded from https://gnomad.broadinstitute.org/downloads#v4-constraint. The SFARI ASD-linked genes were downloaded from https://gene.sfari.org/. The SCZ-linked genes were downloaded from https://www.nature.com/articles/s41586-022-04556-w. Protein abundance measurements in the postsynaptic density of humans, rhesus macaques, and mice were obtained from the supplemental materials of https://www.nature.com/articles/s41586-023-06542-2. The human population DLPFC single-nucleus RNA-seq data used to compute within human cell type-specific gene expression variation were downloaded from https://brainscope.gersteinlab.org/output-sample-annotated-matrix.html. The human–chimpanzee hybrid cortical organoid data were downloaded from https://www.ncbi.nlm.nih.gov/geo/query/acc.cgi?acc=GSE144825. dN/dS estimates for the human and chimp lineages were downloaded from https://doi.org/10.1186/1471-2164-15-599. All code needed to reproduce the analyses described in this study is available at https://github.com/astarr97/Cell_Type_Evolution.
